# Efficacy of a Novel Exoskeletal Robot for Locomotor Rehabilitation in Stroke Patients: A Multi-center, Non-inferiority, Randomized Controlled Trial

**DOI:** 10.3389/fnagi.2021.706569

**Published:** 2021-08-23

**Authors:** Yongqiang Li, Tao Fan, Qi Qi, Jun Wang, Huaide Qiu, Lingye Zhang, Xixi Wu, Jing Ye, Gong Chen, Jianjun Long, Yulong Wang, Guozhi Huang, Jianan Li

**Affiliations:** ^1^Center of Rehabilitation Medicine, The First Affiliated Hospital of Nanjing Medical University, Nanjing, China; ^2^Department of Rehabilitation Medicine, Zhujiang Hospital, Southern Medical University, Guangzhou, China; ^3^Shanghai YangZhi Rehabilitation Hospital, (Shanghai Sunshine Rehabilitation Center), Shanghai, China; ^4^Guangdong Work Injury Rehabilitation Hospital (Guangdong Work Injury Rehabilitation Center), Guangzhou, China; ^5^Jiangsu Zhongshan Geriatric Rehabilitation Hospital, Nanjing, China; ^6^Shenzhen MileBot Robotics Co., Ltd., Shenzhen, China; ^7^Department of Rehabilitation Medicine, The Second People’s Hospital of Shenzhen, Shenzhen, China

**Keywords:** lower-limb exoskeletal rehabilitation robot, stroke, locomotor training, locomotor function, rehabilitation

## Abstract

**Objective**: To investigate the efficacy and safety of a novel lower-limb exoskeletal robot, BEAR-H1 (Shenzhen Milebot Robot Technology), in the locomotor function of subacute stroke patients.

**Methods**: The present study was approved by the ethical committee of the First Affiliated Hospital of Nanjing Medical University (No. 2019-MD-43), and registration was recorded on the Chinese Clinical Trial Registry with a unique identifier: ChiCTR2100044475. A total of 130 patients within 6 months of stroke were randomly divided into two groups: the robot group and the control group. The control group received routine training for walking, while in the robot group, BEAR-H1 lower-limb exoskeletal robot was used for locomotor training. Both groups received two sessions daily, 5 days a week for 4 weeks consecutively. Each session lasted 30 min. Before treatment, after treatment for 2 weeks, and 4 weeks, the patients were assessed based on the 6-minute walking test (6MWT), functional ambulation scale (FAC), Fugl-Meyer assessment lower-limb subscale (FMA-LE), and Vicon gait analysis.

**Results**: After a 4-week intervention, the results of 6MWT, FMA-LE, FAC, cadence, and gait cycle in the two groups significantly improved (*P* < 0.05), but there was no significant difference between the two groups (*P* > 0.05). The ratio of stance phase to that of swing phase, swing phase symmetry ratio (SPSR), and step length symmetry ratio (SLSR) was not significantly improved after 4 weeks of training in both the groups. Further analyses revealed that the robot group exhibited potential benefits, as the point estimates of 6MWT and Δ6MWT (post-pre) at 4 weeks were higher than those in the control group. Additionally, within-group comparison showed that patients in the robot group had a significant improvement in 6MWT earlier than their counterparts in the control group.

**Conclusions**: The rehabilitation robot in this study could improve the locomotor function of stroke patients; however, its effect was no better than conventional locomotor training.

## Introduction

Stroke is the leading cause of long-term disability in adults (GBD 2019 Diseases and Injuries Collaborators, 2020; Virani et al., 2020). With an aging population and advances in emergency care, the prevalence of stroke is increasing annually, leading to significant medical and social burdens (Wang et al., 2017). Previous studies show that up to 90% of stroke patients live with some form of dysfunction, among which locomotor impairment is highly prevalent (Gresham et al., 1975; Mayor, 2015). Asymmetric gait pattern, lower-limb spasticity on the hemiplegic side, as well as compromised ability of single stance and weight shift are observed in most stroke patients, thereby limiting their locomotor function (Lam and Luttmann, 2009). Although with early surgical/pharmaceutical interventions and rehabilitation therapies, 65–85% of the patients manage to walk independently within 6 months post-stroke; however, impaired gait and cardiopulmonary endurance continue to limit the daily ambulation for stroke patients (Shankaranarayana et al., 2021).

Conventional rehabilitation therapies for post-stroke locomotor training are performed manually by multiple therapists. This is labor-consuming, inefficient, and expensive. Besides, the therapeutic effects are subject to the personal skills of therapists and hence homogeneous and standardized therapies are not available for the patients. Additionally, for patients with lower-limb spasticity, at least two therapists are required to complete a training session. Thus, the training doses for individual patients are limited. Previous studies show that stroke registries in mainland China offered approximately 1.43 million physical therapy sessions in 2017; meanwhile, 5.5 million patients are diagnosed with stroke annually and hence there is a large unmet demand for physical therapy (Wang et al., 2019). To bridge this gap in rehabilitation therapies and ensure training doses for stroke survivors, the development and validation of intelligent rehabilitation robots in clinical settings are of great importance.

In recent years, studies recommending the use of exoskeletons in gait training for stroke patients are emerging (Tefertiller et al., 2011; Mehrholz and Pohl, 2012; Pennycott et al., 2012). Parallelly, clinical trials also show the therapeutic benefits of exoskeletons in balance and locomotion (Yeung et al., 2018; Kim et al., 2019; Ii et al., 2020; Moucheboeuf et al., 2020). For example, The ReWalk (ReWalk Robotics, Israel) provides targeted assistance of both paretic ankle plantarflexion and dorsiflexion in overground walking for patients with stroke (Awad et al., 2020). Likewise, gait performance in patients with chronic stroke using Ekso (Ekso Bionics, USA; Calabrò et al., 2018) and HAL (Hybrid Assistive Limb, Japan) is higher as compared to conventional training (Watanabe et al., 2017); gait speed and step length improve significantly (Yoshikawa et al., 2017). Additionally, researchers are using soft wearable robots for transmitting mechanical power generated by off-board or body-worn actuators to the paretic ankle, which can overcome deficits in forward propulsion on the hemiplegic side, thereby improving gait symmetry and reducing the metabolic cost (Awad et al., 2017). However, these soft wearable robots are still in an early stage of development, and validation clinical trials are few. However, only a few studies report the effectiveness of domestically made exoskeletons in China. Thus, the present study aimed at investigating the effectiveness of a domestically made robotic exoskeleton, BEAR-H1, in locomotor rehabilitation in post-stroke cases.

## Materials and Methods

### Ethical Approval and Patient Recruitment

The present study was approved by the ethical committee of the First Affiliated Hospital of Nanjing Medical University (No. 2019-MD-43). Registration was recorded on the Chinese Clinical Trial Registry with a unique identifier: ChiCTR2100044475. All the subjects signed the consent form. Participants were recruited from March 2019 to June 2020 in five rehabilitation centers: the First Affiliated Hospital of Nanjing Medical University, Zhujiang Hospital affiliated to Southern Medical University, Shenzhen Second People’s Hospital, Guangdong Work Injury Rehabilitation Hospital, and Shanghai Sunshine Rehabilitation Center. Patient eligibility criteria were as follows: (1) 18–75 years of age; (2) weight ≤85 kg, height: 1.55–1.90 m; (3) stable vital signs; (4) confirmed diagnosis of a first-ever hemiplegic stroke with duration ranging from 2 weeks to 6 months; (5) upper-limb strength enough to hold parallel bars; (6) impaired gait stability and speed; (7) acceptable range of motion in the hip and knee joints; (8) ankles could be placed in neutral position passively; and (9) cognitive function sufficient for understanding and participating rehabilitation training. Patients with any of the following criteria were excluded: (1) significantly restricted range of joint motion for walking; (2) unhealed fractures or severe osteoporosis; (3) skin injuries or infection in lower limb area; (4) unstable angina, severe arrhythmia, or other heart diseases; (5) severe chronic obstructive pulmonary disease; (6) untreated deep vein thrombosis; (7) pregnancy or lactation period; (8) poor compliance to the study; (9) other contraindications for locomotor training; and (10) ongoing involvement in other clinical trials.

### Description of the Proposed Exoskeleton Robot

**Figure 1:**
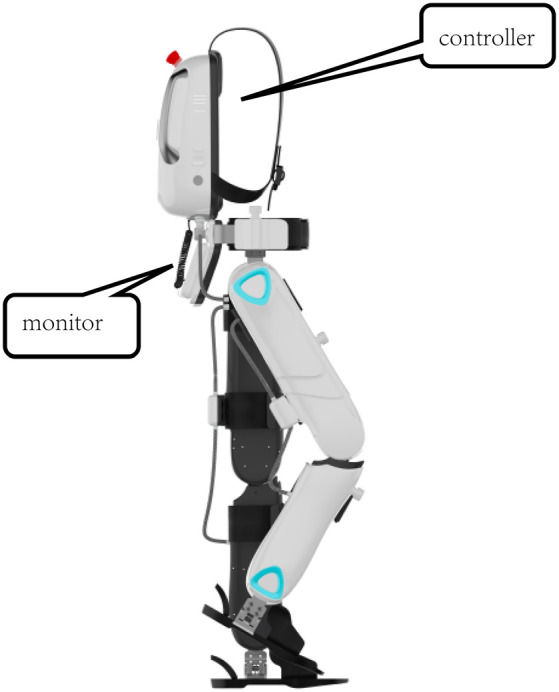
The overall structure of BEAR-H1. The exoskeleton provides assistance with an impedance controller, where the assistance is bassed on the deviation of the joint to the reference trajectory. It is equipped with a gait monitoring and evaluation system, which enables physical therapists to monitor the patient’s motion on the screen in real-time.

The BEAR-H1 (Shenzhen Milebot Robot Technology, Figure 1) was driven by brushless direct current motors to achieve assisted hip flexion/extension, knee flexion/extension, and plantar flexion/ dorsiflexion. It was also equipped with highly accurate sensors, anthropomorphic joints, controllers, and a software system. The software system recognized the patient’s gait through intelligent algorithms based on the collected angle trajectory, human-computer interaction torque, plantar pressure, and other information of the lower limb joints (hip, knee, ankle). The joint configuration of BEAR-H1 was approximately consistent with the human lower limb joints. It had three active degrees of freedom (DOFs) and a passive DOF in each leg. The three DOFs were rotations along the hip joint, knee joint, and ankle joint in the sagittal plane, separately. The adduction and abduction of the hip joint was the passive DOF.

The BEAR-H1 had a training mode and an intelligent mode. For the training mode, stride frequency could be changed within 3% of the set gait cycle frequency. For the intelligent mode, stride frequency could be adjusted in real-time to achieve synchronization of human-robot interaction. The assistance was provided based on the assist-as-need concept. Specifically, reference trajectories of each joint were generated after the human-robot synchronization was achieved. The exoskeleton provided assistance with an impedance controller, where the assistance was based on the deviation of the joint to the reference trajectory. It was equipped with a gait monitoring and evaluation system, which enabled physical therapists to monitor the patient’s motion on the screen in real-time.

### Study Design, Treatment Protocol, and Evaluation

This study was a multi-center, non-inferiority, randomized controlled trial to investigate the effectiveness of a novel exoskeleton robot. Participants were randomized into an intervention group and a control group in 1:1 ratio. Randomization was done using an online system^1^. Subjects in the robot group were given robot-assisted locomotor training using the BEAR-H1 exoskeleton robot, while patients in the control group received routine walk training with assistance from therapists. Both groups received two sessions daily, 5 days a week for 4 weeks consecutively. Each session lasted 30 min.

Relevant indicators were evaluated before the treatment; after 2 weeks of treatment and 4 weeks’ treatment. The evaluations were as follows: 6-min walk test (6MWT; Agarwala and Salzman, 2020); Fugl-Meyer assessment lower-limb subscale (FAM-LE; Gladstone et al., 2002); Functional ambulation category (FAC) evaluation (Park and An, 2016); Vicon gait analysis (the time ratio of the single stance to the swing period on the affected side, cadence, and gait cycle). Gait symmetry was measured using the swing phase symmetry ratio (SPSR) and step length symmetry ratio (SLSR); these were calculated with ratios of gait metrics on both paretic and non-paretic sides (Guzik et al., 2017; Rozanski et al., 2020). Both indicators for gait symmetry were no less than one with the larger number in the numerator (Guzik et al., 2017). Both SPSR and SLSR were further categorized as improved and not improved; changes from baseline larger than minimal detectable change (MDC) were considered as improved and those less than MDC were not improved. The MDC was 0.26 for SPSR and 0.19 for SLSR (Lewek and Randall, 2011). Gait analysis data was collected only before treatment and after 4 weeks of treatment, while all other evaluations were done before treatment, after 2 weeks’ treatment, and 4 weeks’ treatment. The primary outcome is the improvement of 6-min walk test after 4 weeks’ treatment as opposed to baseline.

### Sample Size Calculation

The standard deviation (SD) for the 6-min walking test was set at 15 meters and the non-inferior cut-off was −8 m, based on a previous study (Duncan et al., 2003). With α at 0.025, 1-β at 0.8, and a 10% drop-out rate, the minimal sample size was 128 cases using the following formula:

$$n_{\text{T}}=n_{\text{C}}=\frac{2{\left(Z_{1-\alpha }+Z_{1-\beta }\right)}^{2}\sigma^{2}}{{\left(\left|\mu_{\text{T}}-\mu_{\text{C}}\right|-\Delta \right)}^{2}}$$

*n*_T_: required sample size in the treatment (robot) group;

*n*_C_: required sample size in the control group;

*μ*_T_: mean of the primary outcome in the robot group;

*μ*_C_: mean of the primary outcome in the control group;

*σ*: standard deviation; Δ: non-inferiority cut-off

### Statistical Analyses

Mean and SD were used for continuous data, while median and interquartile intervals were used for ordinal data. The process of patient selection, the actual number of cases in each center, the number of excluded cases, and the number of drop-out cases were recorded, and the intent-to-treat (ITT) along with Per-protocol (PP) sets were defined. For the ITT set, the values of the previous evaluations were used for the missing data so as to avoid overestimation of the treatment effect. *T*-tests were used for normal distribution; otherwise, the rank-sum test was used. Likewise, the Wilcoxon rank-sum test was used for ordinal data comparison. Between-group comparisons for each follow-up point were performed using Analysis of Variance (ANOVA) for normal distribution; else, Kruskal–Wallis tests were used. *P* < 0.05 was considered statistically significant.

## Results

**Figure 2:**
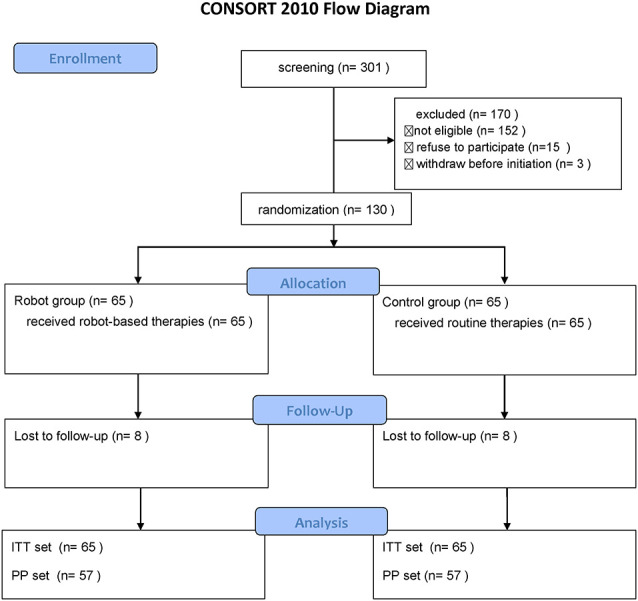
Consort flow diagram of the study.

A total of 130 stroke patients who satisfied the above-mentioned criteria were selected from the centers from March 2019 to June 2020. Patients were randomly divided into the robot and the control groups, with 65 patients in each group. Sixteen patients could not be followed up for personal reasons; a total of 114 patients completed the trial. The enrollment status of patients in each center is shown in Supplementary Table 1, and the overall patient enrollment process is presented in Figure 2.

**Figure 3:**
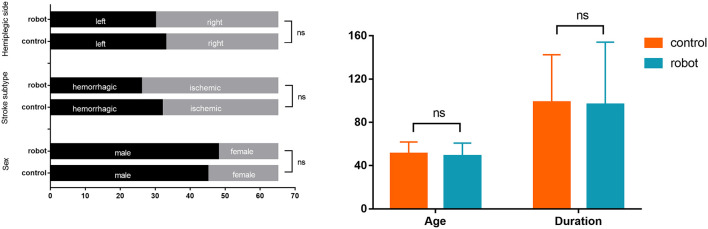
Baseline characteristics of the enrolled subjects. Left panel: categorical data; Right panel: continuous data. Error bars indicate the standard deviation. ns: not significant.

**Figure 4:**
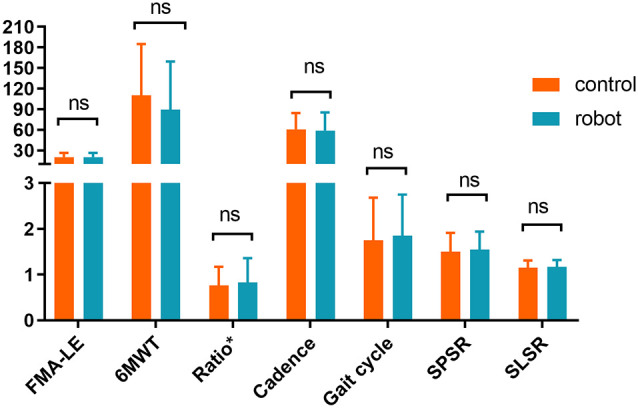
Baseline assessment of the subjects. *Ratio: single stance time vs. swing phase on the affected side. SPSR, swing phase symmetry ratio; SLSR, step length symmetry ratio. Error bar indicates the SD. ns: not significant.

**Figure 5:**
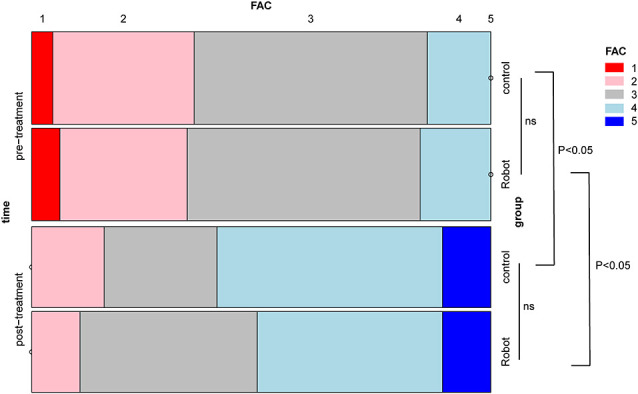
A mosaic plot showing the differences in functional ambulation scale (FAC) scores within the group and between groups. ns: not significant.

There were no significant differences in gender, age, duration of disease, stroke subtype, and hemiplegic side across the two groups of stroke patients (*P* > 0.05; Figure 3). Likewise, no statistically significant differences across the two groups were observed in FMA-LE, 6MWT, the ratio of single stance to the swing phase on the affected side, cadence, and gait cycle at baseline (Figure 4). Indicators on gait symmetry, including SPSR and SLSR, also showed no statistically significant differences across different groups at baseline (Figure 4). Most of the baseline FAC scores of the two groups were distributed at 2–3, and there was no significant difference between groups based on the Wilcoxon rank-sum test (Figure 5). After 4 weeks of treatment, patients in both groups had considerable improvements in FAC score; however, no statistically significant difference was observed between groups.

**Figure 6:**
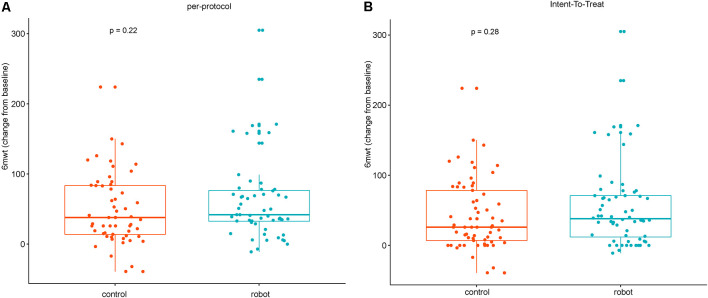
Box plots of the changes in the 6-min walk test (6MWT) between groups after 4 weeks of treatment. **(A)** Per-protocol analysis. **(B)** Intent-to-treat analysis.

As shown in Table 1, the FMA-LE, 6MWT, and cadence of the two groups of patients significantly improved after 4 weeks of treatment as compared to the baseline (*P* < 0.05). Additionally, the gait cycle was also significantly shortened (*P* < 0.05). There was no significant difference in the ratio of the duration of single stance to the swing phase within the group. No inter-group statistical differences were found in the aforementioned indicators. According to the predetermined non-inferiority plan, the null hypothesis was that the 6MWT of the robot group would be smaller than that in the control group, and the difference would exceed 8 m. As shown in Table 1, the point estimate of 6MWT in the robot group was greater than that in the control group, and thus, the null hypothesis was rejected. The changes in the 6MWT between groups were analyzed further. The results indicated that there were no statistical differences in both the PP and the ITT sets (*P* > 0.05). However, the boxplot showed that the mean increase in the 6-min walking distance of patients in the robot group was greater than that of the control group after 4 weeks of treatment (Figure 6). This indicated the potential benefits of robot-based training using the proposed exoskeleton.

**Table 1:** Assessment after 4 weeks of treatment on the subjects ($\bar{x}$ ± s ).

	N	FMA_LE	6MWT	Ratio	Cadence (/min)	Gait cycle (s)
Control	57	23.82 ± 6.7^a^	160.37 ± 101.17^a^	0.75 ± 0.24	73.81 ± 27.25^a^	1.47 ± 0.86^a^
Robot	57	24.44 ± 5.29^a^	150.43 ± 100.77^a^	0.76 ± 0.29	72.17 ± 24.59^a^	1.46 ± 0.8^a^

*Ratio: single stance time vs. swing phase on the affected side; a: significantly improved compared to baseline within group, *P* < 0.05*.

**Figure 7:**
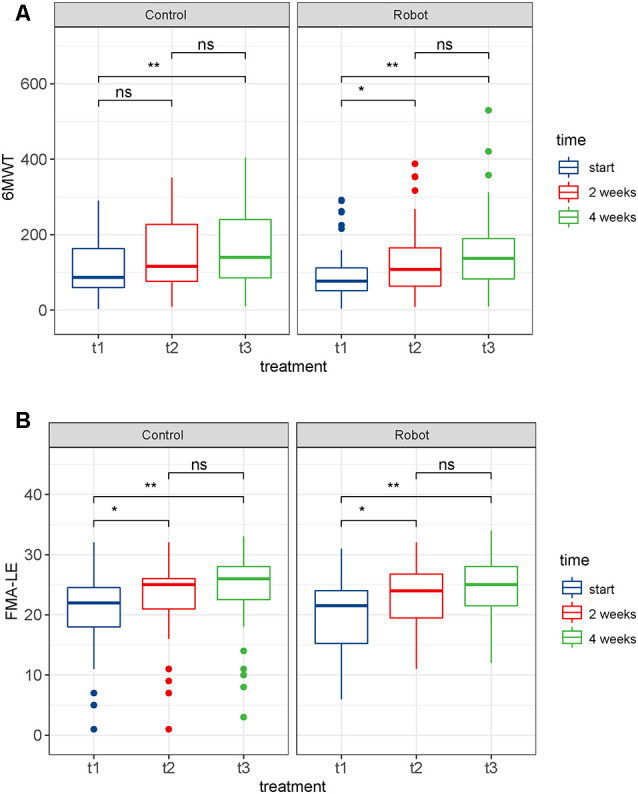
Box plots of the differences in the outcomes at multiple time points. **(A)** 6-min walk test (6MWT). **(B)** Fugl-Meyer assessment lower-limb subscale (FMA-LE). ns: not significant, *p < 0.05, **p < 0.01; t1: before treatment, t2: after 2 weeks of treatment, t3: after 4 weeks of treatment.

Assuming that cases lost due to follow-ups were missing at random, we adopted the PP set to analyze the changes in the continuous variables at multiple time points and the differences between the groups; 6MWT and FMA-LE were analyzed. As shown in Figure 4, there was no statistically significant difference in 6MWT between groups before treatment (t1), after 2 weeks of treatment (t2), and after 4 weeks of treatment (t3; *P* > 0.05). Further analysis of the differences at multiple time points within the group showed that after 2 weeks of routine therapy, there was no significant improvement in 6MWT, while the 2-week robotic therapy was effective statistically (*P* < 0.05). After 4 weeks of treatment, the 6MWT of the two groups of patients was significantly improved as compared to the baseline. However, when compared with 2 weeks’ treatment, there was no significant improvement (*P* > 0.05; Figure 7). Likewise, there was no significant difference in FMA-LE between the groups at the three time points (*P* > 0.05). Further, the two groups of patients showed significant improvement in FMA-LE after 2 weeks of treatment, and this improvement was maintained at 4 weeks; however, there was no significant gain for the additional 2 weeks of treatment (Figure 7).

Analyses of gait symmetry are presented in Table 2. Our findings showed that intra-group differences were not significant on SPSR or SLSR, suggesting no benefits on gait symmetry using either the proposed robot or therapies in the control group for locomotor training within 4 weeks. Between-group differences on gait symmetry indicators were not significant either with the Wilcoxon test or Chi-squared test. However, the proportions of patients showing improvements in gait symmetry in the robot group were higher than that in the control group (SPSR: 35% vs. 30%; SLSR: 16% vs. 12%, respectively).

**Table 2:** Gait symmetry indicators after 4 weeks of treatment on the subjects.

Indicator	Group	*N*	Mean (SD)	Wilcox P	Not improved	Improved*	*χ* ^2^	*χ* ^2^ *P*
SPSR	Control	57	1.4 (0.38)	0.58	40 (70%)	17 (30%)	0.16	0.69
	Robot	57	1.46 (0.36)		37 (65%)	20 (35%)		
SLSR	Control	57	1.1 (0.12)	0.94	50 (88%)	7 (12%)	0.07	0.79
	Robot	57	1.12 (0.13)		48 (84%)	9 (16%)		

SPSR, swing phase symmetry ratio; SLSR, step length symmetry ratio; *if the change of specific indicator was larger than minimal detectable change (MDC), then it was considered Improved; otherwise, it was regarded as Not improved.

## Discussion

In recent years, the exoskeleton robot has been used for locomotor rehabilitation. Its multidisciplinary natures contribute to effective functional compensation or training for individuals with impairments in upper or lower limbs (Pons, 2010; Molteni et al., 2018). In locomotor training, lower-limb exoskeleton robots provide support for patients with insufficient strength to facilitate normal gait (Díaz et al., 2011; Zhong et al., 2020b), offer opportunities for functional recovery with personalized locomotor training programs (Zhang et al., 2017; Shi et al., 2019; Zhong et al., 2020a), and reduce the physical burden of therapists (Díaz et al., 2011). Additionally, exoskeleton-based rehabilitation therapies can objectively and continuously monitor the performance and progress of patients (Louie and Eng, 2016). Even with these merits, clinical validation for most lower-limb exoskeleton robots remain a challenge. The aim of the present study was to validate the effectiveness of an over-ground exoskeleton robot, BEAR-H1. The results of this study showed that the proposed exoskeleton robot effectively improved the patient’s locomotion, lower-limb motor function, and gait parameters. Its effect was equivalent to routine rehabilitation therapies (*P* > 0.05). Further analysis of the 6MWT showed that patients receiving robotic therapy showed higher improvement than with conventional therapies. Specifically, the point estimates of 6MWT at 4 weeks and its changes from baseline in the robot group were greater than that in the control group. In addition, the robot-assisted rehabilitation therapy showed early statistically significant improvement in 6MWT. Consistent with a previous study (Patterson et al., 2015), analysis of gait symmetry revealed no significant improvement on SPSR or SLSR in both groups. Neither was a between-group difference detected on either SPSR or SLSR, indicating no additional benefits of locomotor training using the proposed robot on gait symmetry. However, the proportion of improved individuals for both symmetry indicators in the robot group were higher than that in the control group.

The effectiveness of the exoskeleton robot could be attributed to the following mechanisms: first, bodyweight support of the robot led to increased walking stability and training efficiency of stroke patients (Chua et al., 2020; Pignolo et al., 2020); second, exoskeleton robots provided repetitive, highly intensive, and standardized training with greater continuity and consistency, which contributed to enhanced efficacy (Smith and Thompson, 2008; Langhorne et al., 2011). Additionally, exoskeleton robots promoted blood circulation in the lower limbs and improved cardiopulmonary function, which is in line with a previous clinical trial (Chang et al., 2012). A previous study shows that the 2-week Lokomat robot-assisted training can increase the maximum oxygen uptake (VO2 Max) of stroke patients by up to 12.8%, and also significantly improve the muscle strength of the lower extremities (Chang et al., 2012).

Notably, the patients enrolled in this study were stroke patients in the subacute phase, and the duration of the disease was approximately 3 months. The therapeutic effect may partly be due to natural recovery. With the design of randomized controlled trials, which effectively balanced the natural recovery between groups, the differences in treatment effect between groups could be explained by intervention factors. Previous studies show that the walking function of patients with chronic stroke improves after robot-assisted training (Molteni et al., 2017), suggesting that the effects of robot-assisted locomotor training are independent of spontaneous recovery. Reports suggest that a 4-week robot-assisted locomotor training decreases the lower-limb spasticity and promotes the functional recovery of stroke patients (Cho et al., 2015). Since most of the stroke patients enrolled in this study had mild spasticity (Modified Ashworth Scale: 0–1), there was limited scope for further improvement and no statistically significant differences were observed within the group. Future research on the effects of robot-assisted locomotor training on patients’ lower-limb spasticity should focus on quantifying the degree of lower-limb spasticity using objective indicators. Similarly, robotic therapies to improve gait symmetry in stroke patients warrant further investigation.

The findings of the present study suggested that the proposed lower-limb exoskeleton robot could assist stroke patients in locomotor training with efficacy equivalent to that of conventional therapies. Although the 6MWT of the robot group patients showed a better improvement than conventional treatment, the difference was not statistically significant. Taken together, the advantages of robot-assisted therapy, including a standardized training environment, adaptive support, and sufficient training intensity and doses, the lower limb rehabilitation robot may have implications as a powerful technique for clinical rehabilitation.

## Conclusion

Locomotor training using the proposed exoskeleton robot improved locomotion and lower-limb motor function of stroke patients. However, its effect was equivalent to conventional training. The purpose of introducing robotic therapy in rehabilitation practice is not to replace therapists, but to provide the patients with more choices of safe and effective therapies. The effects of exoskeleton robots in stroke rehabilitation need more investigation.

## Data Availability Statement

The raw data supporting the conclusions of this article will be made available by the authors, without undue reservation.

## Ethics Statement

The studies involving human participants were reviewed and approved by the ethical committee of the first affiliated hospital of Nanjing Medical University (No. 2019-MD-43). The patients/participants provided their written informed consent to participate in this study.

## Author Contributions

YL, TF, QQ, and JW contributed to study design, patient recruitment, and data collection. HQ analyzed the data, wrote the initial draft, and organized the revision with the support of YL. LZ, XW, and JLo contributed to patient recruitment and coordination. JY and GC contributed to the design of the robotic device. GH, YW, and JLi contributed to study design, study supervision, and final approval of the manuscript. All authors contributed to the article and approved the submitted version.

## Conflict of Interest

JY and GC were employed by company Shenzhen MileBot Robotics Co., Ltd. The remaining authors declare that the research was conducted in the absence of any commercial or financial relationships that could be construed as a potential conflict of interest.

## Publisher’s Note

All claims expressed in this article are solely those of the authors and do not necessarily represent those of their affiliated organizations, or those of the publisher, the editors and the reviewers. Any product that may be evaluated in this article, or claim that may be made by its manufacturer, is not guaranteed or endorsed by the publisher.

## Footnotes

^1^ https://pro4.irtone.com/login
